# Longitudinal and Transversal Elasticity of Natural and Artificial Materials for Musical Instrument Reeds

**DOI:** 10.3390/ma13204566

**Published:** 2020-10-14

**Authors:** Enis Ukshini, Joris Jan Jozef Dirckx

**Affiliations:** Department of Physics, Laboratory of Biomedical Physics BIMEF, The University of Antwerp, Groenenborgerlaan 171, 2020 Antwerp, Belgium; joris.dirckx@uantwerpen.be

**Keywords:** elasticity modulus, arundo donax, musical instruments, synthetic reeds, musical acoustics

## Abstract

The reed is the primary component in single-reed woodwind instruments to generate the sound. The airflow of the player’s mouth is the energy source and the airflow is modulated by the reed. The oscillations of the reed control the airflow. Traditionally, instrument reeds are made out of natural cane (Arundo Donax), but in efforts to overcome variability problems, synthetic reeds have been introduced. Previous investigations mainly focused on natural cane reeds and direct elasticity measurements did not discriminate between elasticity moduli along different directions. In order to obtain the mechanical properties along the direction of the reed fibres and in the orthogonal direction separately, a three-point bending testing setup was developed, which accommodates the small samples that can be cut from an instrument reed. Static moduli of elasticity were acquired in both directions. Much higher ratios between longitudinal and transversal moduli were seen in the natural cane reed as compared to the artificial reeds. Wet natural reeds showed a strong decrease in moduli of elasticity as compared to dry reeds. Elasticity was significantly higher in artificial reeds. The force–displacement curves of the wet natural reed show hysteresis, whereas the artificial materials did not. In the cane reed, higher energy losses were found in the transversal direction compared to the longitudinal direction

## 1. Introduction

In single-reed instruments such as the clarinet and saxophone, the source of the sound is formed by periodic motions of the tip of the reed, which is driven into oscillation by an airstream. During playing, the overpressure in the mouth generates an airstream passing through the small slit between the tip of the reed and the mouthpiece. Due to the Bernoulli effect, this air stream causes a pressure difference between the upper and lower surface of the reed. When the flow is high enough, the negative Bernoulli pressure causes the reed to bend further towards the mouthpiece, thus decreasing the flow when air pressure in the mouth is increased. This effect corresponds to a negative hydrodynamic resistance, which is the source of energy to drive the reed in periodic, usually non-harmonic motion. The motion of the reed depends on many parameters, and reed elasticity is one of the prime factors. The vibrations of the reed modulate the airstream, which is the sound source of the instrument. In order to control the vibrations and the acoustic impedance of the mouthpiece (which in turn influences the pitch produced by the instrument), the pressure of the player’s lips on the top side of the reed makes the reed bend towards the mouthpiece, thus changing its mechanical properties. The player aims at forming a specific opening between the tip of the reed and the tip of the mouthpiece while bending the rest of the reed towards the side rails of the mouthpiece to form an airtight fit. The tip of the reed needs to be very light and stiff so that the natural frequency of this spring-mass system is high enough to adapt to all musical tones that can be played on the instrument. So, in the direction along the long axis of the mouthpiece, the elasticity modulus of the reed material needs to be sufficiently high. We will refer to this orientation as the longitudinal direction. In the transversal direction, the reed needs to be flexible enough so that the player’s lips can bend the reed to form the airtight fit with the side rails of the mouthpiece.

Traditionally, these material parameters have been realized by using reeds made out of natural cane, a giant reed grass (Arundo Donax). The Arundo Donax grows to a height of 4–6 meters, and the stem is hollow. The outer diameter is approximately 2–3 cm [1]. The material characteristics of the Arundo Donax are influenced by different growing and harvesting conditions. Climate, soil conditions, and time of harvest are such examples. These factors induce that the reed is the component with by far the most variability compared to the mouthpiece and instrument tube, and it therefore has a substantial influence on the sound of the instrument. The macroscopic structure of the natural cane is made up of three concentric layers [2]. The outer layer consists of hard waxy epidermis and outer cortical cells. The second layer is a thick sclerified fibre which comprises mostly parenchyma cells where the cell wall is thicker (sclerified) than in normal thin-walled parenchyma cells. The inner layer consists of vascular bundles and parenchyma cells [3]. To manufacture a reed, the outer and middle layer of the macroscopic structure of Arundo Donax are removed towards the tip of the reed so that the vibration part consists of the vascular bundles and the parenchyma cells. Those vascular bundles are commonly referred to as the fibre of the reed. The longitudinal direction of the reed is chosen parallel to the cane fibres. When the reed is bent, fibres are stretched, which gives the reed a high stiffness along the longitudinal direction. No transversal fibres are present, so to bend the reed in that direction only the matrix of parenchyma cells needs to be bent, which has a lower elasticity modulus than the fibres.

For many years, reeds have only been produced out of natural cane. Acoustically, this material performs very well, but it also has specific drawbacks. Before starting to play, the reed needs to be wetted, which strongly alters its elasticity properties. Therefore, the shape of the reed is made so that it is aimed to have the desired mechanical properties in wet condition. Prolonged exposure to a wet environment makes the fibres disintegrate, and bacteria in the player’s saliva gradually fill the channels in the reed fibres, thus altering the reeds behaviour. Material fatigue also progresses rather fast, so that the performance of the reed degrades over periods of tens of hours of playing. As cane is a natural material, fibre density and irregularities in fibre orientation differ between reeds, so each change to another reed necessitates an adaptation by the player. For these reasons, many attempts have been made to seek artificial materials that can replace the natural reed. Plastic materials such as polycarbonate or polyacrylate have been used, but with little success. These materials have isotropic material parameters, making them either too soft in one direction or too hard in the other. For about a decade [4], artificial reeds have emerged on the market which contain fibres, giving them strongly anisotropic elasticity parameters comparable to natural reeds. Two leading manufacturers in this field are the Fiberreed [5] (Harry Hartmann’s Fiberreed, 70771 LE-Oberaichen, Leinfelden-Echterdingen, Germany) and the Légère reeds (Légère Reed Ltd., Barrie, Ontario, Canada). Fiberreed consists of a Hollow Fiber Foamresin Compound (HFC), and Légère reeds are made of a homogeneous material consisting of polypropylene fibres (US6087571).

The elasticity of cane reeds has been measured in previous studies using indentation testing [6]. This test method has the advantage that the test can be performed on tiny samples. However, the test does not give information on the anisotropic elasticity values separately in the two principal directions of the reed. A limited number of papers report on elasticity parameters of natural reed materials. In [7,8], the longitudinal elasticity modulus of natural cane was measured, and values between 4 and 7 GPa are reported. Taillard et al. [9] pointed out the importance of the bending of the reed in the direction perpendicular to its axis, and presented modelling results using different mechanical parameters in the directions along and perpendicular to the length axis of the reed.

In the current paper, we will investigate the anisotropic elasticity parameters of both artificial materials (Carbon Fiberreed and Légère Reed) and compare them to wet and dry natural reeds. Natural reeds are produced by many manufacturers, and we will use reeds of one of the world leading companies, Vandoren (Paris, France).

Reeds are produced in different strengths, which mainly refers to the stiffness of the thinned portion and tip of the reed. In natural reeds, the strength can also be influenced by the part of the cane stem where the reed is cut. In the present study, we use material taken from tenor saxophone reeds of strength 3, which is a commonly used average strength.

Because natural reeds are cut out of cane, their size in transversal direction is limited to about 15 mm. We therefore developed a setup that allows us to perform a three-point bending test on reed samples that are just 14 mm in length. Based on this method, we will report in this paper on longitudinal and transversal elasticity of natural and artificial reeds.

## 2. Measurement Setup and Specimen Preparation

**Figure 1:**
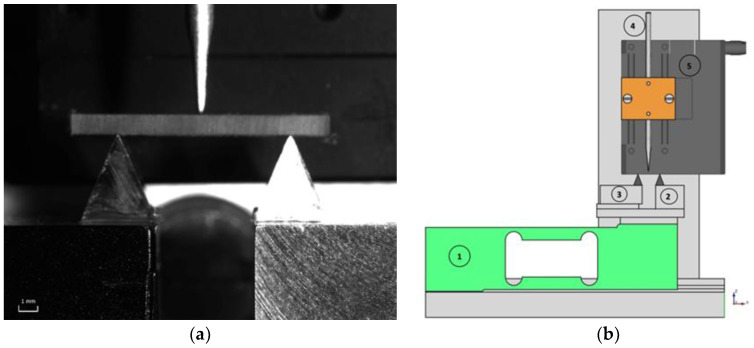
(**a**) Close-up of the support system. (**b**) Schematic drawing of the setup. 1-Load cell, 2-specimen support, 3-horizontal translation stage, 4-indentation stylus, 5-motorized translation stage.

The setup is based on the principle of the three-point bending test performed on simply supported beams. In this test, one support point is fixed while the other can move in the direction perpendicular to the bending. The support that can move horizontally accommodates for the elongation of the bottom side of the specimen. Figure 1a shows a close-up of the support system. A schematic drawing is represented in Figure 1b as well.

**Figure 2:**
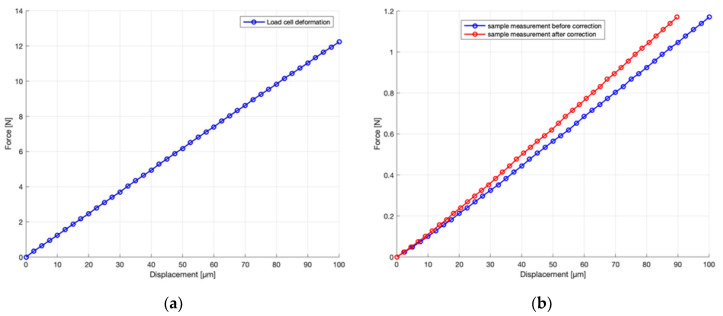
(**a**) Force–displacement curve of the load cell. (**b**) Typical measurement curve before and after correcting for load cell compliance.

The specimen rests on two steel wedges, one of which is mounted on a nearly frictionless translation table. The bending is applied in the centre with a steel stylus, which is connected to a high-resolution translation stage (PI M-112 micro translation stage). The support is mounted on a load cell (Tedea-Huntleigh single point load cell, Model 1042). Since the load cell will have some force-dependent deformation on its own, the slope of the force–displacement curve associated with the measurement setup was determined without placing a test specimen between the supports and the steel stylus. Subsequently, these data were used to correct the applied stylus movement to true specimen deformation by subtracting the load cell deformation from the applied stylus displacement for each measured force value. Figure 2a shows the load-deformation curve of the load cell and Figure 2b shows a sample measurement before and after correcting using the slope of the force–displacement curve associated with the measurement setup. It can be seen in Figure 2a that the slope is almost perfectly linear. For deformations larger than 10 micrometres, the deviations of the fitted curve compared to the measured curve are smaller than 1%. For displacements lower than 10 micrometres, deviations up to 2% were measured. The load cell output signal was digitized and stored in the computer during the loading and unloading cycle. Data recording started as soon as the force transducer signal reached 1.5 mV (conversion factor 1 mV = 1 mN), which is just above the system noise floor. Due to the measurement noise, the cycle going down sometimes ends at voltages lower than 1.5 mV, which is occasionally seen on the graphs as a small negative force. Obviously, such negative force does not exist in reality, and the value falls within the noise interval around zero. The sampling rate was set to 100 kHz, and the acquisition time was 100 ms. The same computer controlled the translation stage with an accuracy of better than 10 nanometres. This assured that the displacement was applied without shocks. When the motion of the stage is reversed at the end of the cycle, the stage has a mechanical backlash of approximately 2 micrometres. Because of this inevitable mechanical limitation, data points at the end of the bending cycle will not be used in calculations of modulus of elasticity.

Specimens of 14 mm length, 1 mm thickness, and 2 mm width were prepared by cutting samples out of the thick end part of the reeds, flattening, and polishing them as well as possible to the desired dimensions. As thickness has an important influence on the elasticity values obtained from a three-point bending test (power to the third), the obtained dimensions were measured with a calliper with an accuracy of 10 micrometres. The actual thickness varied between 0.88 and 0.99 mm amongst samples. The samples were tested in the custom-made setup using three subsequent bending cycles of 100 micrometres, at intervals of 2.5 micrometres. This deformation range ensured that the specimens were not permanently deformed in order to determine the elastic properties of the reeds. Between each bending step, the specimen was left to relax for 0.1 seconds to avoid viscoelasticity effects. One full indentation and release cycle takes 86 seconds. The specimens of the natural reeds were drained in water during 15 minutes. A test was done on specimens drained for several hours, and no measurable changes were found. A high-resolution calibrated camera (2056 × 2464 pixels) was used to follow the bending process, and to determine the distance between the support points with an accuracy of 11 micrometres.

From the measurement data, the modulus of elasticity *E* was calculated according to the Euler–Bernoulli equation:

(1) $$E=\frac{Fl^{3}}{48Iw}$$

in which w is the deflection, *F* is the force, l is the distance between the supports, and *I* is the second moment of area of the specimen. *I* is calculated from *I* = $\frac{bh^{3}}{12}$, with b the width of the specimen and *h* the height. The slope of the force-displacement curve determined the Young’s modulus of the reed specimens.

The modulus was determined over intervals along the bending cycle. As a trade-off between resolution and noise, we used intervals of approximately 10 micrometres of bending yielding five measurement points along the bending trajectory. Over the intervals, the curve was approximated by linear interpolation of the recorded force data. Elasticity was calculated separately for the loading and unloading part of the cycle. 

**Figure 3:**
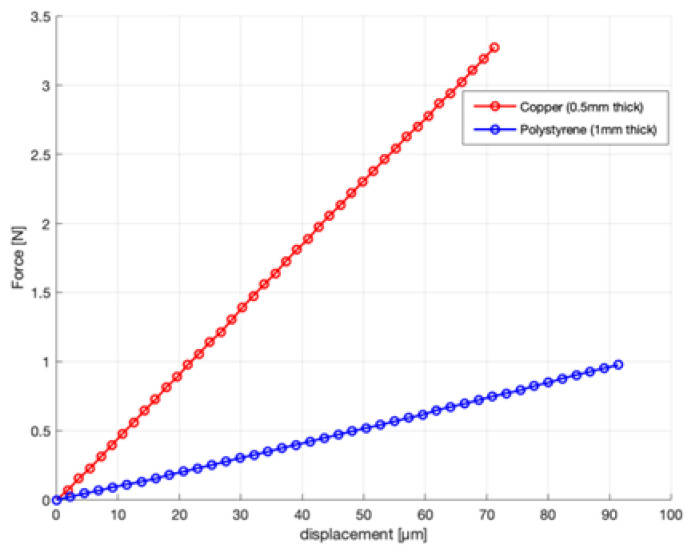
Force–displacement curve of a copper 0.5 mm sample, polystyrene 1.5 mm sample, and polystyrene 1.5 mm sample, obtained in the three-point bending test.

To validate the setup and method, measurements were performed on a 1.0 mm thick sample of polystyrene and a 0.5 mm thick sample of copper with a span length of 14 mm. For copper, we used a thinner sample to stay within the force range of the setup. The loading curves are shown in Figure 3. For copper, we found an elasticity modulus of 121 GPa, with a literature value between 117 GPa and 133 GPa [10,11,12].

According to the ASTM D143 standard, the thickness-to-length ratio of samples in a three-point bending test should be minimal 1:14 to get good results. The length of the reed samples is limited to 14 mm, so we tried to do all measurements on samples of 1 mm thickness. For some experiments, we had to use thicker samples to obtain force values that were high enough to get reliable measurements. We will come back to this in the discussion. For the polystyrene sample of 1.0 mm thickness, we found a value of 3.1 GPa. This is in the range reported in literature, which varies between 3 and 3.5 GPa [13].

As the actual dimensions of the specimens differ slightly from the desired dimensions, we will report the measured force data after normalizing everything to 1 mm thickness and 2 mm width, according to the linear dependence of bending force on the width and the power to the third dependence on thickness.

## 3. Results

Table 1 gives an overview of the actual dimensions of the specimens. The length between the support points was 13.6 mm. The mean thickness and width were calculated from three measurements across the geometry of the specimen. Since the thickness and width of the moisturized reeds will change due to the swelling, the wet specimens were measured again instantly after the draining in water.

**Figure 4:**
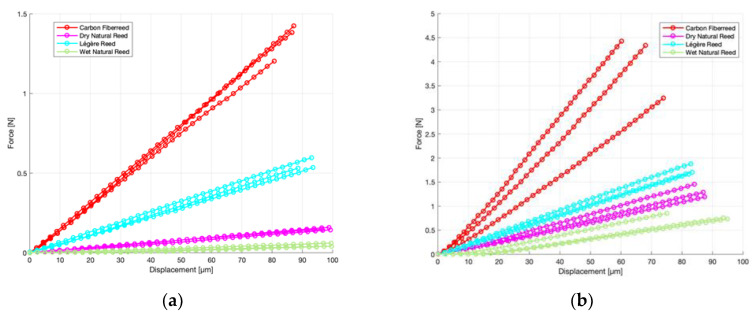
(**a**) Force–displacement curves of different reed materials in transversal direction. (**b**) Force–displacement curves of different reed materials in longitudinal direction.

To compare the different materials and specimens with each other, preconditioning of the specimens was needed so that the measured force-displacement curves became repeatable. For the wet natural reed, the difference between the first and second cycle is large. After the second cycle, differences between the cycles were less than 1% for most measurements from 10 micrometres onwards. Deviations between cycles are far smaller than the differences between the materials and directions seen in Figure 4 below. Therefore, the final third bending cycle is shown for all the specimens.

Figure 4 shows the results of the loading curves obtained in the third bending cycles for all four materials (purple: Dry natural cane; green: Wet natural cane, red: Carbon Fiberreed; blue: Legère reed) in the longitudinal and transversal direction. The different curves for each material are the three different specimens.

The results show that the measured force in the longitudinal direction was for all four materials larger than the transversal direction showing the anisotropic properties of the different materials. Table 2 shows the mean normalized (thickness: 1 mm; width: 2 mm) force at 40 micrometres. The force for the Carbon Fiberreed specimens in the longitudinal direction was 2.27 N with a deviation of 0.6 N or more than 25%. Since the Carbon Fiberreed specimens are layered, the amount of carbon layers has a substantial influence on the measured force and stiffness of the specimen.

We noticed that the Carbon Fiberreed longitudinal specimen with the lowest measured force had one layer of carbon less compared to the other two specimens. The force increased linearly. In the transversal direction, the deviation between the Carbon Fiberreed samples was small (0.02 N) compared to the mean force value of 0.62 N.

The longitudinal elasticity of Légère specimens is in the same order of magnitude as the longitudinal elasticity of the dry reed samples. The mean force at 40 micrometres for the Légère longitudinal reed samples was 0.85 N, whereas 0.57 N for the dry reed specimens. The slope of the longitudinal Légère specimens’ curve is also more in trend with the longitudinal natural reed specimens compared to the longitudinal Carbon Fiberreed samples. The measured normalized force for the transversal Légère specimens was 0.24 N and 0.07 N for the transversal dry reed samples.

For the wet natural reed specimens, the measured normalized values at 40 micrometres were 0.015 N for the transversal direction and 0.28 N for the longitudinal direction. Due to the natural character of the reed and the low measured values for the force, deviations were high, at. 0.008 N and 0.1 N, respectively. Wetting of the natural reed specimens has a much higher impact in the transversal direction than the longitudinal direction. In the longitudinal direction, the force values decreased with a factor of approximately two whereas in the transversal direction, a decrease with a factor of more than five was observed.

To compare the different slopes of the force–displacement curves of the materials, we calculated the elasticity modulus of each measured specimen from the slope of the loading curve. The results are also shown in Table 2. In the longitudinal direction, the Carbon Fiberreed had the highest modulus of elasticity. A mean value of 18.9 GPa was found with a high standard deviation of 6.4 GPa, probably mainly caused by the different number of carbon layers in different samples. When comparing this value to the natural dry reed, it is seen that the slope of the Carbon Fiberreed was much steeper than the natural dry reed. The mean Young’s modulus of the dry reed in the longitudinal direction was 5.0 (±0.7) GPa or more than three times less stiff compared to the longitudinal Carbon Fiberreed specimen. When the natural reed was drained in water, the Young’s modulus in the longitudinal direction decreased to 3.1 (±0.4) GPa. The longitudinal Légère specimens had an elasticity modulus of 7.5 (±0.3) GPa. This is in good trend, seen by the slopes from Figure 4, with the elasticity modulus of the natural dry longitudinal specimens. Nevertheless, this value is more than two times higher compared to the wet longitudinal reed Young’s modulus. Generally, the natural wet reed is the most flexible material resulting in the lowest Young’s modulus in both directions.

Furthermore, in the transversal direction, the Carbon Fiberreed had the highest modulus of elasticity at 5.3 (±0.3) GPa. Subsequently, the plastic Légère reeds had the second-highest elasticity modulus with a value of 1.9 (±0.1) GPa. When comparing those values to the dry natural reed, the Carbon Fiberreed and plastic Légère reed were a factor of 10.6 and 3.8 stiffer in the transversal direction. The Young’s modulus of dry natural reed was 0.5 (±0.03) GPa. Nevertheless, when the natural reed was drained in water, the Young’s modulus decreased to 0.1 (±0.05) GPa. As a result, the Carbon Fiberreed and plastic Légère reed were now more than a factor of 50 and 19 stiffer in the transversal direction.

The ratio between the longitudinal and transversal elasticity moduli is given in Table 2. For the Légère reeds, the ratio is 4.0. A comparable value of 3.6 is found for the Carbon Fiber reeds. A much higher value of 10.0 was found for the natural dry reeds, and this ratio becomes much higher when the reeds are wet. The standard deviation of the ratio longitudinal/transversal of the wet reed is higher (24.3 (±8.0)), so the wettening process increases variability between reeds.

**Figure 5:**
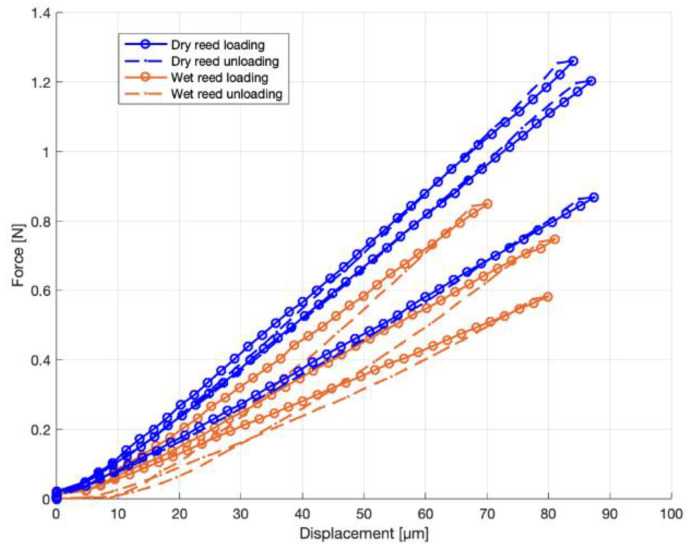
Force–displacement curves of dry and wet reed samples in the longitudinal direction.

No noticeable differences between the loading and unloading curve were found in the Carbon Fiberreed and plastic Légère reeds. For the natural reeds, the loading and unloading curves did not coincide, which indicated the visco-elastic properties of the natural cane. Figure 5 shows the force–displacement curves of the longitudinal reed samples. The blue curves represent the dry reed specimens. The brown/orange curves show the measurement of wet reed specimens. The full line is the forward bending part of the cycle and the dashed line represents the relaxation part. It is seen that the loading and unloading curve of the dry reed specimens coincided when taking into account the mechanical backlash at the top of the curves. However, for the wet reed specimens, hysteresis was clearly visible.

In the wet natural reeds, elastic strain energy is dissipated in the wet cane material. Since we measure the force with the corresponding displacement, the enclosed surface area represents the energy loss in µJ. Table 3 summarizes the energy losses for each measured specimen calculated from the third bending cycle. Energy losses are given as a percentage as well to enable a relative comparison since differences in maximum displacement were noticed between specimens.

To be able to better measure the hysteresis of the wet reeds, specimens with a thickness of 1.5 mm were also tested. For these specimens, hysteresis was apparently noticeable for both the longitudinal and transversal direction. The corresponding values are reported in Table 3. The energy loss is calculated by firstly determining the surface under the loading curve and hereafter subtracting the surface under the unloading curve. For the thicker specimens a higher energy loss was seen in the longitudinal direction. The energy loss raised from 15.7% for the 1 mm high specimens to 24.1% for the 1.5 mm thick specimens. When comparing the energy losses in the transversal direction to the longitudinal direction, it was observed that more energy (33.3%) is dissipated in the transversal direction.

All the loading and unloading curves of the tested specimens showed a nearly linear behaviour, except the wet reed specimens. For the wet reed specimens, the measured points showed a larger deviation from a linear fit in the unloading curve in the [0, 10] µm range where the closing of the hysteresis occurred.

When the mean elasticity modulus was calculated for the wet longitudinal specimens, the values for the three different samples were, respectively, 3.36 GPa, 3.38 GPa, and 2.61 GPa for the loading curve and 3.44 GPa, 3.45 GPa, and 2.58 GPa for the unloading curve, so the moduli of elasticity are nearly the same for loading and unloading. Furthermore, no trend was seen between the stiffness of the reed specimens and the amount of losses due to visco-elasticity.

For the wet natural reeds, we needed to use samples of 1.5 mm thickness to obtain large enough force values for reliable measurements. As we will discuss later, this leads to an artefact. To have a better view on this effect, we also tested the other reed materials on samples with 1.5 mm thickness. The elasticity calculations for the specimens with different thicknesses are shown in Table 4. In the longitudinal direction, higher elasticity moduli were found when the thickness of the samples was reduced. In the transversal direction, the effect is limited to less than 20% for the artificial materials and for dry cane. For wet cane the effect is nearly 60%. In the longitudinal direction, the effect was, respectively, 22.7% and 27.1% for the Carbon Fiberreed and the Légère reed. For wet cane, the effect was 47.6% and for dry cane, 66.7%. In general, reducing the thickness of the specimens had a larger influence in the longitudinal direction compared to the transversal direction, and was more pronounced on the natural material.

## 4. Discussion

### 4.1. Methodology

The reed materials used for testing were only available in limited size, so a special setup needed to be developed to do a three-point bending test on such small samples. The aspect ratio of the specimens needs to fulfil certain criteria so that the correct desired stress–strain rate is induced from the applied boundary conditions. The elasticity modulus can be underestimated by the three-point bending test due to the shear forces and the indentation effect of the loading head and supports [14]. Since the influence of the shear stresses is highly influenced by the span-to-height ratio, a span-to-height ratio of a minimum of 14 needs to be ensured, according to the ASTM D143 for static bending testing [15]. When the samples were cut to 1.5 mm, this criterion was not fulfilled, and a lower modulus of elasticity was measured. On the other hand, forces are higher for the thicker samples so they can be measured with better accuracy. For this reason, we reduced our samples to less than 1 mm at the given maximal length of 14 mm so that influence of shear stresses was minimized. Only for the measurements on wet natural reeds in transversal direction did the 1.5 mm samples prove to be necessary to obtain better signal to noise ratio in the force measurement.

The recording of the force–displacement curves was triggered when the output voltage of the strain gage amplifier reached 1.5 mV, corresponding to a force of 1.5 mN. The maximum displacement was set to 100 micrometres to assure the samples were elastically deformed and not damaged. A difficulty in the mechanical setup was the positioning of the samples between the supports. A high-resolution camera was used to observe the positioning of the sample so that it could be aligned with the supports to minimize torsional effects and to assure that the steel stylus was perfectly perpendicular on the surface specimen. We verified with the copper and polystyrene samples that, despite remaining inaccuracies, reliable results were obtained that correspond to literature values.

### 4.2. Experimental Results

No literature values of modulus of elasticity were available for the Carbon Fiberreeds. The force-displacement curves in Figure 4 showed that the Carbon Fiberreed samples had the highest stiffness. Besides the Hollow Fiber Foamresin Compound (HFC) material, the Carbon Fiberreed also contained carbon fibres mimicking the fibres of the natural cane. The observed high stiffness in the current study is more due to the carbon fibres than HFC since moduli of elasticity of reinforced carbon are high [16]. Besides this, the elasticity modulus of carbon fibre materials is highly dependent on the composite material used. In the longitudinal direction, the stiffness of the Carbon Fiberreed is six times higher than the stiffness of wet natural cane, while in the transversal direction, it is more than 50 times stiffer. Moreover, the Carbon Fiberreeds were non-homogeneous due to the alternating layers of carbon and HFC. In [17], it is described that only the last one-quarter to three-eighths of an inch at the tip of the reed controls the elastic response. Remarkably, no carbon fibres were observed in this region so that the contribution to the elastic response and longitudinal stiffness of the reed is minimal when played. Only the HFC material was seen at the reed tip.

For the polypropylene reed, the patent of Légère reports that a synthetic reed with an oriented semi-crystalline thermoplastic material has been made with the longitudinal modulus and density the same as cane in playing conditions. At the beginning of the patent, it is reported that isotropic polypropylene with a density of approximately 0.91 g/mL has an elastic modulus of approximately 1.0 to 1.6 GPa, less than one-third the modulus of cane. It is not known how, for the patent, the modulus of elasticity was determined for both polypropylene and cane. A longitudinal modulus of a conditioned reed of 5-10 GPa is mentioned further on in the patent. Our results show a longitudinal elasticity modulus of 7.5 GPa for the Légère reeds, which lies between the reported values of 5–10 GPa, and a transversal modulus of 1.9 GPa. Compared to the 3.1 GPa found in the longitudinal direction of wet cane, the Légère reed material comes the closest to the properties of the natural material. In the transversal direction however, the Légère material (1.9 GPa) is nearly 20 times stiffer than wet natural cane (0.1 GPa). For the transversal direction, no values for modulus of elasticity are given in [4] for the Légère reeds, but the patent does state that the transversal modulus of the Légère polypropylene reed is considerably higher compared to the transversal elasticity modulus of the natural cane.

More literature data can be found for natural cane. In [1], the Young’s modulus on hollow cylinders were determined of the hypodermal sterome or the outer layer of the cane in the longitudinal direction from which the parenchyma cells were removed. Values of approximately 10 GPa were found in the lower and middle part of the stem. These values decreased in the high part of the stem. The Young’s modulus of the parenchyma cells was determined as well by subtracting the contribution of the hypodermal sterome from the overall stiffness, which was measured by a three-point bending test. The average longitudinal Young’s modulus was 9 GPa in the middle of the stem and reduced significantly in the upper part of the stem (values of 1–2 GPa). The experiments of the current study show an average modulus of elasticity of 5.0 ± 0.7 GPa in the longitudinal direction for the natural dry reed which is in the measured range reported by Spatz et al. [1]. In a more recent study of the same authors [18], the modulus of elasticity was obtained for seven natural cane plants with no leaves in the base part. The authors compared the results acquired from their experiment to the results of a three-point bending test on an independent set from the same clonal stand of the Arundo Donax (H. Beissner, unpublished data). The average values were 4.79 ± 0.7 GPa and 5.23 ± 1.25 GPa which are in trend with our results obtained on small samples. Weidenfeller et al. [8] compared the storage modulus of two Arundo Donax samples by DMA and found a value of 5.25 GPa and 6.25 GPa. Although the static modulus of elasticity is similar conceptually to the storage modulus, values will differ. Nonetheless, the loss factor was small at low frequencies in order for the difference between the storage and elastic modulus to be low.

Soaking the natural cane samples in water reduced the modulus of elasticity both in longitudinal and transversal directions. Especially in the transversal direction, the effect was huge: Elasticity dropped from 0.5 GPa to 0.1 GPa, while in the longitudinal direction, it decreased from 5.0 GPa to 3.1 GPa. Obataya et al. [19] studied the effect of relative humidity (RH) on the Arundo Donax and showed that the dynamic Young’s modulus decreased with increasing the RH as well. In future, more insight in the macroscopic and microscopic elastic behaviour of reed material could be obtained using ultrasonic techniques to generate longitudinal waves. From the wave propagation velocity, elasticity parameters could be calculated not only in longitudinal and transversal directions of the specimens, but also along the thickness axis.

It is likely that the low elasticity modulus in the transversal direction is related to a better sealing to the mouthpiece as it will help the player to easily bend the sides of the reed with the lips to make a tight seal with the mouthpiece. Open sides lead to the so-called, and feared, “squeak” sound, especially in less-practiced players. Also, the very low transversal modulus can lead to an effective de-coupling between vibrating fibres, which in turn may have a huge effect on vibrational modes of the reed tip when playing the instrument. In wet natural cane, the ratio of longitudinal over transversal modulus of elasticity is a factor of more than 24. In the artificial materials, this ratio is approximately 4. The difference can be compensated by using different thickness profiles, but a technical problem can occur when thickness needs to be reduced so far as to obtain stiffness values that approximate the natural reeds.

The purpose of the loading-unloading curves was to estimate hysteresis effects caused by visco-elasticity. Dissipation of energy due to visco-elasticity has been reported in Arundo Donax [20]. The current measurements showed a significant difference in visco-elasticity between dry and wet reed samples. The hysteresis increased when the reeds were soaked in water. This is advantageous since visco-elasticity in wood materials dampens the vibrations [21], thus leading to less sharp resonance peaks in the vibration spectrum.

## 5. Conclusions

The elastic material parameters were investigated for natural and artificial reeds in the two principal directions of the reed. A setup was designed to evaluate the moduli of elasticity on small reed specimens by a three-point bending test. A comparison of the longitudinal moduli of elasticity to the transversal bending moduli indicates that currently used instrument reed materials are anisotropic. The highest moduli of elasticity were found for the Carbon Fiberreed with mean values of 18.9 GPa in the longitudinal direction and 5.3 GPa in the transversal direction. For the plastic Légère reeds, mean values of 7.5 GPa and 1.9 GPa were obtained. These values were higher than those of natural dry reed, but the values measured on wet natural reeds, mimicking the playing condition, are still far lower, especially in transversal direction, with values of 3.1 GPa in the longitudinal and 0.1 GPa in the transversal direction. The ratio of the longitudinal/transversal modulus of elasticity was about 24 for the wet natural reed specimens, which is much higher than for any of the other materials. No visco-elasticity was observed in the synthetic reed materials and natural dry reed, but it was clearly present in the natural wet reed specimens. The results showed that energy losses were higher in the transversal direction compared to the longitudinal direction.

## Figures and Tables

**Table 1:** Thickness and width of different specimens.

Specimen Name	Mean Thickness (mm)	Mean Width (mm)
Carbon Fiberreed 1 Transversal	0.95 ± 0.01	1.91 ± 0.02
Carbon Fiberreed 1 Longitudinal	0.95 ± 0.02	1.92 ± 0.01
Légère Reed 1 Transversal	0.95 ± 0.01	2.01 ± 0.01
Légère Reed 1 Longitudinal	0.95 ± 0.01	1.93 ± 0.02
Dry Natural Reed 1 Transversal	0.95 ± 0.02	2.05 ± 0.01
Dry Natural Reed 1 Longitudinal	0.97 ± 0.01	1.93 ± 0.03
Wet Natural Reed 1 Transversal	0.98 ± 0.02	2.04 ± 0.01
Wet Natural Reed 1 Longitudinal	0.99 ± 0.02	1.96 ± 0.02
Carbon Fiberreed 2 Transversal	0.95 ± 0.01	1.95 ± 0.02
Carbon Fiberreed 2 Longitudinal	0.97 ± 0.02	1.90 ± 0.02
Légère Reed 2 Transversal	0.95 ± 0.01	2.00 ± 0.01
Légère Reed 2 Longitudinal	0.96 ± 0.01	1.86 ± 0.02
Dry Natural Reed 2 Transversal	0.88 ± 0.03	2.02 ± 0.01
Dry Natural Reed 2 Longitudinal	0.95 ± 0.02	1.94 ± 0.02
Wet Natural Reed 2 Transversal	0.99 ± 0.02	1.94 ± 0.01
Wet Natural Reed 2 Longitudinal	0.99 ± 0.02	1.94 ± 0.02
Carbon Fiberreed 3 Transversal	0.93 ± 0.03	1.97 ± 0.03
Carbon Fiberreed 3 Longitudinal	0.92 ± 0.01	2.00 ± 0.01
Légère Reed 3 Transversal	0.95 ± 0.01	2.01 ± 0.02
Légère Reed 3 Longitudinal	0.94 ± 0.02	1.89 ± 0.03
Dry Natural Reed 3 Transversal	0.94 ± 0.01	2.00 ± 0.02
Dry Natural Reed 3 Longitudinal	0.91 ± 0.03	2.00 ± 0.02
Wet Natural Reed 3 Transversal	0.95 ± 0.01	2.00 ± 0.02
Wet Natural Reed 3 Longitudinal	0.93 ± 0.02	2.03 ± 0.02

**Table 2:** Summary of the measured force and moduli of elasticity for the different reed materials.

	Force at 40 μm Transversal (N)	Force at 40 μm Longitudinal (N)	Ratio Longitudinal/Transversal	Young’s Modulus Transversal (GPa)	Young’s Modulus Longitudinal (GPa)
Carbon Fibereed	0.62 (±0.02)	2.27 (±0.6)	3.6 (±1.5)	5.3 (±0.3)	18.9 (±6.4)
Légère Reed	0.24 (±0.02)	0.85 (±0.06)	4.0 (±0.3)	1.9 (±0.1)	7.5 (±0.3)
Dry Reed	0.07 (±0.002)	0.57 (±0.07)	10.0 (±2.1)	0.5 (±0.03)	5.0 (±0.7)
Wet Reed	0.015 (±0.008)	0.28 (±0.1)	24.3 (±8.0)	0.1 (±0.05)	3.1 (±0.4)

**Table 3:** Hysteresis losses for different samples of wet natural reed.

	Longitudinal Thickness 1.5 mm		Transversal Thickness 1.5 mm		Longitudinal Thickness 1.0 mm	
	Energy Loss [µJ]	Energy Loss [%]	Energy Loss [µJ]	Energy Loss [%]	Energy Loss [µJ]	Energy Loss [%]
Sample 1	7.3	14.2	1.6	30.6	4.7	19.1
Sample 2	11.7	32.8	1.2	28.1	3.9	16.1
Sample 3	11.2	25.3	1.8	41.2	2.3	11.9

**Table 4:** Moduli of elasticity for reed specimens with different thickness.

	1.5 mm Young’s Modulus Transversal (GPa)	1 mm Young’s Modulus Transversal (GPa)	Difference (%)	1.5 mm Young’s Modulus Longitudinal (GPa)	1mm Young’s Modulus Longitudinal (GPa)	Difference (%)
Carbon Fiberreed	4.3 (±0.3)	5.3 (±0.3)	19.1	15.4 (±1.1)	18.9 (±6.4)	22.7
Légère Reed	1.8 (±0.01)	1.9 (±0.1)	7.7	5.9 (±0.2)	7.5 (±0.3)	27.1
Dry Reed	0.4 (±0.06)	0.5 (±0.03)	11.6	3.0 (±0.2)	5.0 (±0.7)	66.7
Wet Reed	0.2 (±0.02)	0.1 (±0.05)	59.5	2.1 (±0.3)	3.1 (±0.4)	47.6
